# The Salinity Responsive Mechanism of a Hydroxyproline-Tolerant Mutant of Peanut Based on Digital Gene Expression Profiling Analysis

**DOI:** 10.1371/journal.pone.0162556

**Published:** 2016-09-23

**Authors:** Jiongming Sui, Defeng Jiang, Dandan Zhang, Xiaojun Song, Jingshan Wang, Mingxia Zhao, Lixian Qiao

**Affiliations:** 1 College of Life Science, Qingdao Agricultural University, Key Laboratory of Qingdao Major Crop Germplasm Resource Innovation and Application, Key Lab of Plant Biotechnology in Universities of Shandong, Qingdao 266109, China; 2 College of Agronomy and Plant Protection, Qingdao Agricultural University, Qingdao 266109, China; Estacion Experimental del Zaidin, SPAIN

## Abstract

Soil salinity seriously limits plant growth and yield. Strategies have been developed for plants to cope with various environmental stresses during evolution. To screen for the broad-spectrum genes and the molecular mechanism about a hydroxyproline-tolerant mutant of peanut with enhanced salinity resistance under salinity stress, digital gene expression (DGE) sequencing was performed in the leaves of salinity-resistant mutant (S2) and Huayu20 as control (S4) under salt stress. The results indicate that major transcription factor families linked to salinity stress responses (NAC, bHLH, WRKY, AP2/ERF) are differentially expressed in the leaves of peanut under salinity stress. In addition, genes related to cell wall loosening and stiffening (xyloglucan endotransglucosylase/hydrolases, peroxidases, lipid transfer protein, expansin, extension), late embryogenesis abundant protein family, fatty acid biosynthesis and metabolism (13-lipoxygenase omega-6 fatty acid desaturase, omega-3 fatty acid desaturase) and some previously reported stress-related genes encoding proteins such as defensin, universal stress protein, metallothionein, peroxidase etc, and some other known or unknown function stress related genes, have been identified. The information from this study will be useful for further research on the mechanism of salinity resistance and will provide a useful genomic resource for the breeding of salinity resistance variety in peanut.

## Introduction

Peanut (*Arachis hypogaea* L.) is an important crop for oil and protein production in the tropical and subtropical regions of the world [1]. It is very likely for it to suffer from salinity stress which would seriously limit its growth and production in the future. Therefore, it is of great importance to identify the salinity stress resistant genes on a large scale and develop a better understanding of peanut’s salt tolerance. Unfortunately, little progress has been made in this field and one important reason is the lack of germplasm with high resistance to salinity stress [2].

In our previous studies, *in vitro* mutagenesis with pingyangmycin as the mutagen and directed screening with medium containing hydroxyproline (HYP) was conducted to generate mutants of peanuts. The M_3_ generation individuals of regenerated plants were subjected to stress treatment at the seedling stages and the activities of peroxidase (POD) and superoxide dismutase (SOD) increased substantially in eight offsprings of 11 HYP-tolerant, regenerated plants compared with that in their mutagenic parents [3]. As an osmolyte, proline plays an important role in salt tolerance, drought resistance and other physiological processes [4]. The proline analog HYP has been used in tissue culture to select for stress resistant cell lines for many crops [5–6]. In a germination test with a 0.7% NaCl solution, a HYP-tolerant mutant with enhanced salinity-tolerance was obtained and the germination rate of the seeds from the mutant (about 50%) was substantially higher than that of the seeds from the mutagenic parent, Huayu 20 (7.5%). It can inherit stably after selfing and grow better in the saline-alkali field in Dongying city of China. However, very little is currently known on the molecular mechanisms of peanuts which are salt tolerant.

A series of physiological and biochemical strategies have been developed for plants to cope with environmental stresses during evolution, such as through abscisic acid (ABA) and calcium functioning as signals to regulate the expression of transcriptional factor or downstream molecules cascades. The accumulation of organic compatible solutes including sugars, amino acids, betaines and ectoines, low-weight protective proteins including late embryogenesis abundant proteins (LEAs), heat shock proteins (HSPs) and low temperature induced protein. The regulation of ion uptake, change of photosynthesis pathway, expression of major intrinsic proteins or aquaporins, antioxidant defense system including peroxidase, superoxide dismutase and hydrogen peroxidase, and change on structure of cell wall as well [7].

To further illustrate how the salinity-resistant mutant works, with global investment, a comparison between the transcriptome profile in the leaves of salinity-resistant mutant and the wild group under salinity stress was conducted. Following the salinity stress treatment, the dynamic discrepancies of transcriptome profiles were recorded and analyzed and the specific transcripts for salinity stress resistance were identified. The potential roles of differentially expressed genes (DEGs) were discussed and the salinity stress resistance mechanism of this salinity-resistant mutant of peanut was also explored.

## Materials and Methods

### Plant stress treatments and measurement of SOD, POD and MDA

The seeds of salinity-resistant mutant (S2) and the wild Huayu 20 (S4), were planted in 15cm-diameter pots full of sandy soil, with 3000Lux light intensity 11dark/13light cycle, and the temperature being 28°C. Six-week-old, seedlings of S2 and S4 were irrigated with 250 mM NaCl for salinity stress treatment. Forty-eight hours later, the leaves were collected from S2 and S4, and the activities of SOD and POD were measured by the Nitro-blue tetrazolium and guaiacol colorimetric method respectively [8–9]. In addition, the content of MDA was measured by thiobarbituric acid method [10]. Each determination included three replications.

### Library construction for transcriptome sequencing and functional annotation

The leaves samples at the time points of 0, 6, 12, 24 and 48 h of S2 and S4 were prepared and RNA were isolated by means of Trizol reagent (Invitrogen, US), then an equivalent amount of RNA at each point was pooled and analyzed with Agilent 2100 Bioanalyzer (Agilent Technologies, Santa Clara, CA). RNA-sequencing was conducted by Novogene Bioinformatics Technology Co., Ltd (Beijing, China) based on illumina sequencing on a HiSeq2000 system. Next, 125-bp paired-end reads were generated, all clean sequence read data were deposited in the NCBI SRA database (accession number SRR3114511), and assembled into comprehensive unigenes by means of Trinity and TGICL. Functional annotation of genes was conducted based on the databases of NR, NT, SwissProt, PFAM, KOG, GO and KEGG (unpublished data).

### DGE sequencing and mapping

The samples of RNA taken from S2 and S4 were numbered in accordance with the sampling time points of 0, 6, 12, 24 and 48 h respectively. These tested samples were referred to as S2_0, S2_6, S2_12, S2_24 and S2_48 for S2 and S4_0, S4_6, S4_12, S4_24 and S4_48 for S4 respectively, with each treatment involving two similar replicates.

DGE sequencing was conducted by Novogene Institute based on illumina sequencing on a HiSeq2000 system with each reaction having a single 50-bp end read. All clean sequence read data were deposited in the NCBI SRA database (accession number SRR3210665 and SRR3210666). All reads of each library were separately mapped onto the unigenes, and unigene expression was normalized with the value of RPKM (reads per kilobase per million reads).

### Identification of DEGs

Multi-dimensional comparisons were made between the data sets of different samples. The criteria that the adjusted p-value was under 0.05 was adopted as the threshold to evaluate the significance of gene expression difference. Expression was compared both within each genotype and between the two genotypes. The comparison between S2 and S4 samples resulted in D series data sets, which represented the DEGs between S2 and S4 samples in response to salinity stress treatment, and they were denoted as D_0, D_6, D_12, D_24 and D_48 with the sampling time points of 0, 6, 12, 24 and 48 h respectively. Between the genotypes, expression was compared at 0, 6, 12, 24, and 48 h. If the level of expression was significantly different (the adjusted p value < 0.05) in a comparison, the gene would be considered to be differentially expressed. Within S2 and S4, expression was compared between each two sampling time of 0, 6, 12, 24, and 48 h, if the level of expression was significantly up- or down-regulated (the adjusted p value < 0.05) in a comparison, this gene was proposed to be responsive to salinity stress. Pathways that were statistically significant (FDR≤0.05) were enriched with KEGG.

### RT-qPCR analysis

The reverse transcripts were carried out with an Invitrogen SuperScript Reagent Kit. The primer was designed by Oligo6 software. For RT-qPCR, the SYBR^®^ Premix Ex Taq^™^ (TAKARA) was adopted on a Bio-Rad CFX96 real-time PCR detection system (Bio-Rad, Hercules, CA). Gene expression of the samples of S2 and S4 at 6, 12, 24 and 48 h after the salinity stress was analyzed. The relative expression level of each gene among the samples was computed with the 2^-ΔΔCt^ method with normalization to the internal reference genes *Actin* and *TUA5* of peanut, which has been reported to have stable expression in leaves when subjected to salt stress treatment [11]. The relative expression level of each gene in roots at 6, 12, 24 and 48 h after the salt stress had also been analyzed and confirmed, with normalization to the internal reference genes *Actin* and *HDC* of peanut, which has been reported to have stable expression in roots when subjected to salt stress treatment [11]. All reactions of each gene were conducted with three biological replicates respectively. The parameters of thermal cycle were 95°C for 30 s, followed by 40 cycles of 95°C for 10 s, 50–56°C for 25 s with the volume being 20 μl.

### Cis-elements responsive to salt stress in upper promoter

Some genes which could respond to salt stress treatment were blasted in the database whose access website is http://www.peanutbase.org, and the sequence which had highest similarity was regarded as candidate gene. The functional prediction on cis-elements related was conducted by Plantcare (http://bioinformatics.psb.ugent.be/webtools/plantcare/html/) in about 2000 bp possible upper promoter sequence.

## Results

### Activities of SOD and POD and content of MDA

The activities of SOD and POD in S2 was up to 168.2 U/g/min and 1627.3 U/g/min respectively, which was significantly higher than the value 123.4 U/g/min and 1209.2 U/g/min in S4 at 48h after salt stress treatment. Besides, the content of MDA in S2 was up to 19.5 nmol/g/min, and this was significantly lower than the value 39.0 nmol/g/min in S4 at 48h after salt stress treatment.

### Digital gene expression library sequencing and mapping

The quantity of unigenes (FPKM>0.3) in S2 ranged from 49, 022 to 51, 986 with a value of 51,054 on average, whereas the quantity of unigenes (FPKM>0.3) in S4 ranged from 48,713 to 53,742 with a value of 50, 849 on average. About 88.72% to 90.84% of the clean reads in each sample were mapped to our transcriptome reference database (S1 File).

### Clustering analysis of samples and identification of DEGs

**Fig 1:**
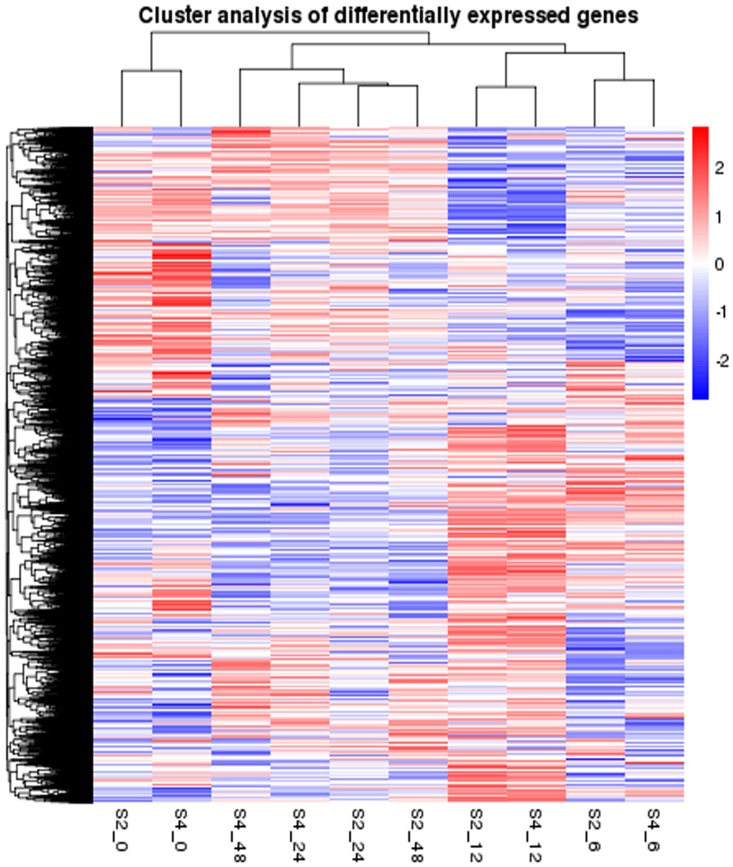
Hierarchical clustering analysis of salinity-induced changes in gene expression in leaves of peanut. S2 is the salinity-tolerant mutant, and S4 is Huayu 20 (control); the first letter plus number refers to the S2 or S4 sample and the second number refers to time points before and after salinity stress treatment. The value of each treatment refers to an average value of two different replications.

To get an overall view, the gene expression profiling for the ten samples from these two genotypes of S2 and S4 were analyzed by means of clustering algorithms and treeview. Apart from the samples at 48 h, the other ones at the same time point displayed quite similar gene expression patterns (Fig 1).

In current studies, after multi-dimensional comparisons, the DEGs were identified under the standard that the adjusted p-value was under 0.05. The variant comparisons between the control group and treated samples exposed to salinity stress were displayed in the S2 and S4 series data sets. These ten series data sets represented the DEGs before and after treatment by salinity stress. The comparison between S2 and S4 samples generated D series data sets, which represented the DEGs between S2 and S4 samples responsive to salinity stress treatment.

**Fig 2:**
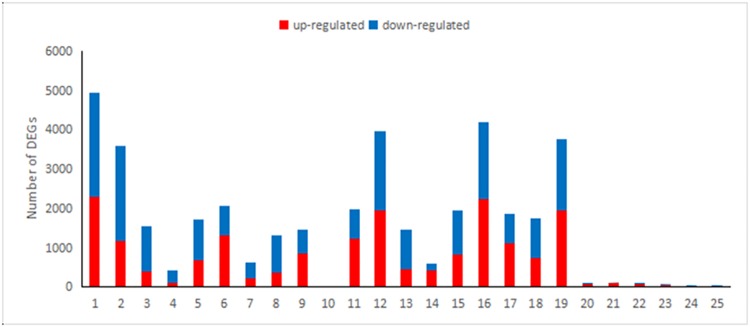
Changes in DGEs during 250 mmol/L NaCl stress in the salinity resistant mutant (S2), control (S4) and D series data sets. The Y axis represents number of DEGs, data sets 1–10 represent S2_6vsS2_0, S2_12vsS2_0, S2_12vsS2_6, S2_24 vsS2_0, S2_24vsS2_6, S2_24vsS2_12, S2_48vsS2_0, S2_48vsS2_6, S2_48 vsS2_12, S2_48vsS2_24; data sets 11–20 represent S4_6vsS4_0, S4_12vsS4_0, S4_12vsS4_6, S4_24vsS4_0, S4_24vsS4_6, S4_24vsS4_12, S4_48vsS4_0, S4_48vsS4_6, S4_48vsS4_12, S4_48vsS4_24; data sets 21–25 represent D_0, D_6, D_12, D_24, D_48. Orange indicates up-regulation, and blue indicates down-regulation.

**Fig 3:**
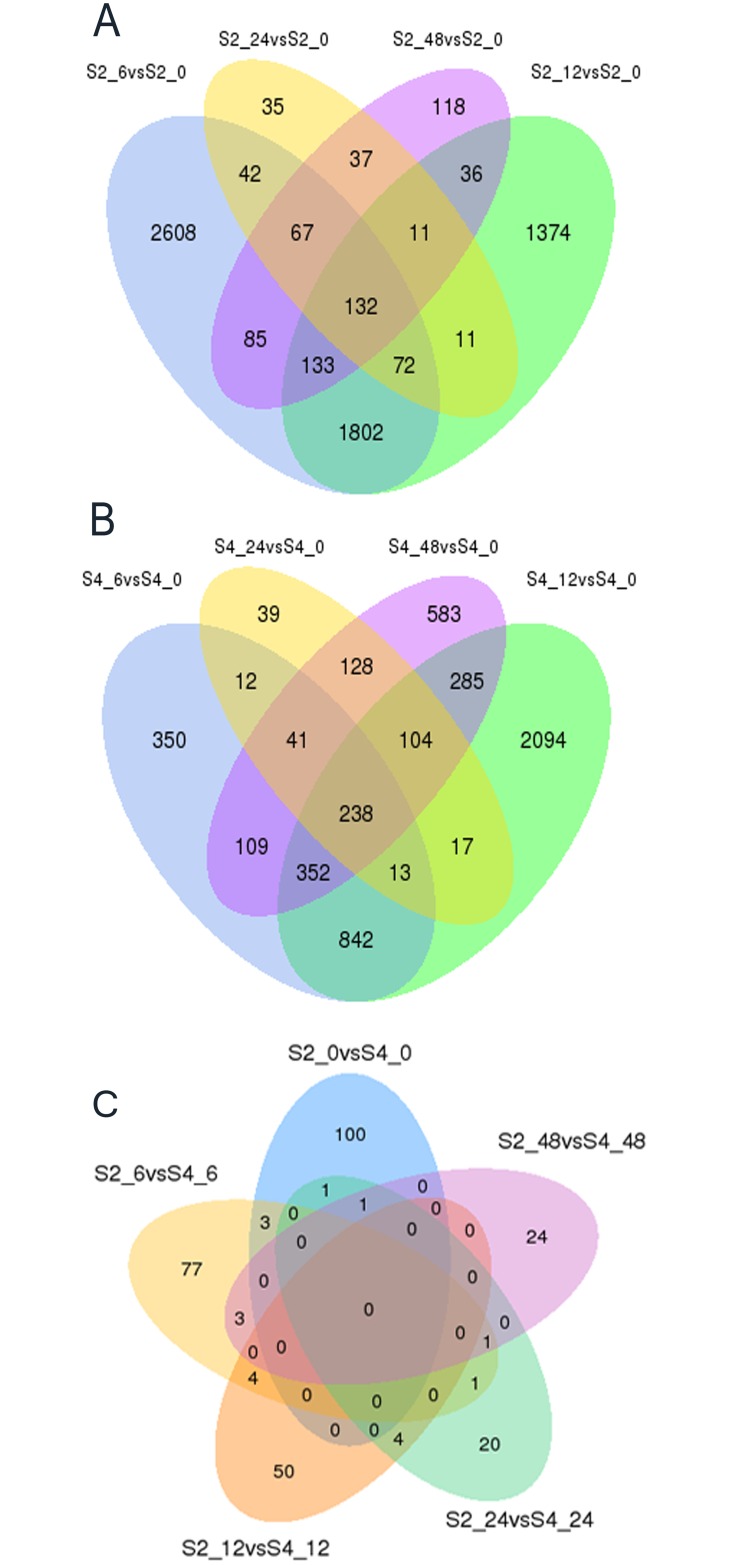
Comparison between the amount of DEGs found in S2 (A), S4 (B) and D (C) series data sets. The venn diagram depicts the number of statistically significant DEGs. The number of DEGs exclusively expressed in one sample is shown in each circle. The number of DEGs with a common tendency of expression changes between the two treatments is shown in the overlapping regions.

The dynamic trends of DEGs in S2, S4 and D data sets were explored. Remarkable changes of gene expression profiling were observed. In comparison with the control group at 0 h, the genes of 1319, 2131, 114 and 361 were in response to salinity stress at the different time points of 6, 12, 24 and 48 h in both S2 and S4 respectively (Fig 2). There were 132 DEGs which were the same at the above-mentioned four different time points after salinity stress treatment in S2, while 238 DEGs were found in common at the four different time points in S4 (Fig 3a and 3b).

For the D series data sets, a peak appeared at 6 h in all the up-regulated and down-regulated DEGs and the number of up-regulated DEGs was remarkably higher than those down-regulated at 6 and 12 h and lower at 24 and 48 h (Fig 2). D_24 and D_48 possessed less exclusive DEGs, whereas D_0 and D_6 possessed most exclusive DEGs. What's interesting is, 18 DEGs appeared in their neighboring data sets (Fig 3c).

### The annotation of DEGs

**Fig 4:**
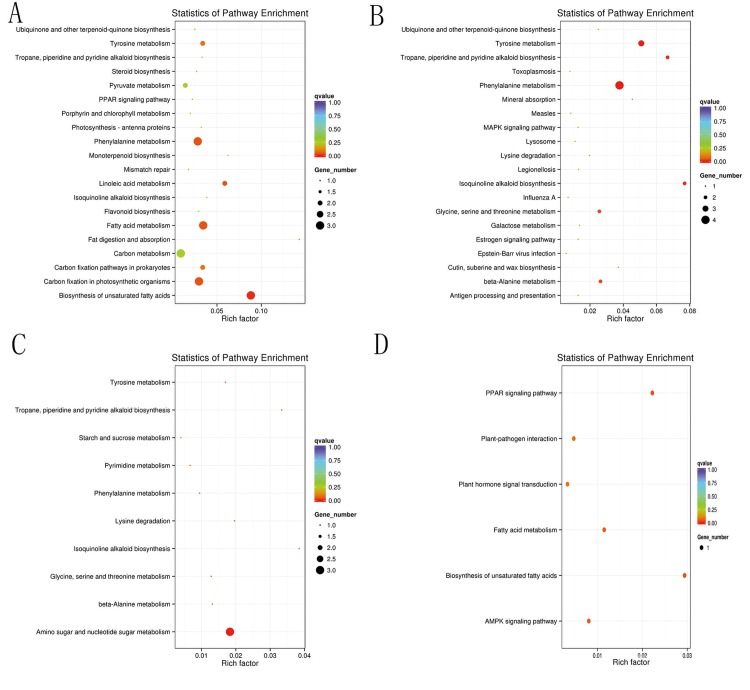
KEGG Pathway enrichment analyses of DEGs in D_6 (a), D_12 (b), D_24 (c) and D_48 (d) series data sets.

For functional annotation of DEGs, the KEGG enrichment analyses were mainly referenced. For D series data sets, according to the corrected p-value, eleven DEGs with down-regulation and three DEGs with up-regulation at 6 h were assigned KEGG pathways and the main concerned pathways were shown in Fig 4a, among which one DEG (c33086_g1) encoding glutathione S-transferase was found to be involved in a few pathways including “Chemical carcinogenesis”, “Drug metabolism-cytochrome P450” and “Glutathione metabolism”. Interestingly, The enrichment analyses indicated that all of the DEGs involved in “Biosynthesis of unsaturated fatty acids / fatty acid metabolism” (3/3) and “Starch and sucrose metabolism” (2/2) were up-regulated, while the more dominant expression pattern of down-regulation was observed on the DEGs involved in “Phenylalanine metabolism”(3/3), “Linoleic acid metabolism”(2/2), “Carbon fixation pathways in prokaryotes”(2/3), “Tyrosine metabolism”(2/2) and “Pyruvate metabolism”(2/2).

Eleven DEGs with down-regulation and three DEGs with up-regulation at 12 h were assigned KEGG pathways, and the main of which were shown in Fig 4b. Interestingly, two down-regulated DEGs (c42886_g1 and c28953_g1) were found to be involved in “Phenylalanine metabolism”, “Tyrosine metabolism”, “Isoquinoline alkaloid biosynthesis”, “Tropane, piperidine and pyridine alkaloid biosynthesis”, “beta-Alanine metabolism” and “Glycine, serine and threonine metabolism”. One DEG (c33729_g1) which encoded molecular chaperones HSP70 was involved in many pathways including “Endocytosis”, “Antigen processing and presentation, “Spliceosome”, “MAPK signaling pathway” and “Protein processing in endoplasmic reticulum”. Another DEG (c36329_g1) which encoded 4-hydroxyphenylpyruvate dioxygenase was down-regulated at both 6 h and 12 h.

Five DEGs with down-regulation and two DEGs with up-regulation at 24 h were assigned KEGG pathways (Fig 4c). Among them, one DEG (c28953_g1),down-regulated at both 12 h and 24 h, was involved in many pathways including “Isoquinoline alkaloid biosynthesis”, “Tropane, piperidine and pyridine alkaloid biosynthesis”, “Tyrosine metabolism” and “Glycine, serine and threonine metabolism”.

A total of 29 DEGs were screened out after salinity stress of 48 h, but only three of them were assigned KEGG pathways (Fig 4d). Among them, one DEG (c33027_g1) which was up-regulated at 6 and 48 h was found to be involved in “Biosynthesis of unsaturated fatty acids”, “Fatty acid metabolism”, “PPAR signaling pathway” and “AMPK signaling pathway”. Another DEG (c36899_g1) which encoded protein phosphatase 2C was involved in “Plant hormone signaling transduction”.

### Screening of salinity responsive genes in D data sets

To further elucidate the salinity resistant mechanism of the HYP-tolerant mutant, those genes whose expression were significantly up or down regulated at 6, 12, 24 and 48 h after salinity stress treatment in D data sets were selected, and a total of 189 DEGs were screened (S2 File). The expression patterns of the genes taking key roles in plant resistance to salinity stress were investigated. Among them, 166 DEGs were responsive to salinity stress treatment both in S2 and/or S4 (S3 File): 103 DEGs which were responsive to salinity stress treatment both in S2 and in S4 were identified, and 27 DEGs which were responsive to salinity stress treatment only in S2 genotype were observed. In the S4 genotype, 36 DEGs which were responsive to salinity stress treatment were detected with significant differences. Interestingly, 17 of the 18 DEGs in D data sets which occurred in the neighboring data sets were responsive to salinity stress treatment.

### Experimental verification of DEGs

To verify the reliability of salinity-responsive genes, we monitored the expression pattern of ten candidate DEGs at the five time points before and after salinity stress for both genotypes with RT-qPCR. These candidate DEGs included genes that were proved to be related to stress response in other plant species. The relative expression levels of the 10 candidate genes were measured before and after salt stress treatment with normalization to the internal reference genes *Actin* and *TUA5* of peanut, which has been reported to have stable expression in leaves when subjected to salt stress treatment [11]. Their relative expression levels calculated according to two different reference genes *Actin* and *TUA5* showed basically linear correlation to the results of the RNA-seq (S1 Fig, S4 and S5 Files). The relative expression levels of the 10 candidate genes in roots were also measured before and after salt stress treatment with normalization to the internal reference genes *Actin* and *HDC* of peanut, which has been reported to have stable expression in roots when subjected to salt stress treatment [11]. The result showed that the relative expression levels of the ten candidate genes in roots were differently regulated when subjected to salt stress treatment (S2 Fig, S5 File). The primers of selected genes and reference genes were listed in S6 File.

## Discussion

### Transcription factors (TFs) in relation to salinity stress

The DGE analysis showed that in all the 8 TFs including AP2/ERF, bZIP, NAC, WRKY and bHLH, zinc finger family were differentially expressed under salinity stress between S2 and S4 (D data sets).

NAC transcription factors belong to a unique class of transcription factors in plants. Extensive studies have revealed that NAC transcription factors not only play important roles in plant growth and development, they also function in regulation of responses to biotic and abiotic stresses [12–13]. A NAC transcriptional factor from peanut (AhNAC2) was isolated and over-expressed in *Arabidopsis*. The transgenic plants exhibited enhanced tolerance to drought and salinity stress, and the *AhNAC2* gene was identified to be a major player in the NAC family involved in ABA signaling [13]. Four NAC TFs (c38875_g1, c40119_g2, c24761_g1 and c40119_g3) were all down-regulated in the D_6 or (and) D_48 data sets. The differentially expressed NAC TFs may be involved in the regulation of resistance to salinity stress in the salinity-resistant mutant of peanut.

WRKY TFs have been found to play key roles in both the repression and derepression of responses to salt stress as well as other abiotic stresses [14]. Overexpression of *OsWRK45* and *OsWRKY72* enhanced salinity and drought tolerance of transgenic plants [15–16]. OsWRK3, which might simultaneously play roles in multiple stress induced signaling pathways, was found to negatively regulate rice tolerance to salinity and cold stresses but positively regulate disease resistance [17]. One WRKY TF (c26767_g2) was down- regulated in D_48 data set in our experiment.

In this study, a bHLH-type TF (c31710_g1) was found to be up-regulated in the D_6 data set. Zahaf et al (2012) has demonstrated that bHLH-type TFs are linked to the adaptation of *Medicago truncatula* to saline soil environments [18]. Another research indicated that the bHLH TF modulated the ROS balance by directly regulating the expression of a set of peroxidases [19]. While a peroxidase (c13583_g1) was down-regulated in the D_6 data set in our experiment. This observation indicated that the bHLH TF might be linked to the expression of the peroxidase in our salinity-resistant mutant.

Otherwise, one ethylene-responsive AP2/ERF TF (c32320_g1) was found to be responsive to salinity stress. AP2/ERF TFs have been proved to be involved in biotic and abiotic stress and plant hormones signal transduction, which are cross-talk factors in all kinds of stress signal pathways [20].

### Identification of DEGs involved in cell wall loosening

The balance between cell wall loosening and stiffening activities defines the regions of accelerated and decelerated growth in the elongation zone of plants. Several proteins including xyloglucan endotransglucosylase/hydrolases (XETs), expansion and extension etc. have directly implicated this balance [21–22]. DGE analyses indicate that the expression of genes related to cell wall loosening and stiffening is modified under salinity stress in the peanut mutant. One XET transcript (c9996_g1) was found to be up-regulated in D_6 data set, while the other two XET transcripts (c39293_g5 and c35569_g1) were down-regulated, especially DEG c39293_g5 being down-regulated at both 24 h and 48 h. In addition to *XET* genes, two expansin transcripts (c19788_g1and c36171_g1) and two extension transcripts (c42238_g1 and c25443_g1) were all up-regulated at 6 h.

Two DEGs encoding lipid transfer protein (c35574_g1 and c25403_g1) were also found to be responsive to salinity stress in D series data sets. Among them, DEG c25403_g1 was down-regulated at 6 h and then up-regulated at 12 h (the Log2 (fold change) was over 4). Plant lipid transfer proteins (LTPs) are a group of abundantly expressed small basic proteins, which can reversibly bind and transport hydrophobic molecules *in vitro*. LTPs are considered to play a role in key processes of plant physiology, including wax synthesis and transport in cell wall, abiotic stress resistance, disease resistance, etc [23–24]. Cameron et al (2008) found that the expression of *LTP* gene was consistent with the wax synthesis under kinds of stressful conditions [23].

### Identification of late embryogenesis abundant protein (LEA)

In plants, a group of very hydrophilic proteins, known as LEA proteins, accumulate to high levels during the last stage of seed maturation and under abiotic stresses in vegetative organs, suggesting a protective role during damage caused by environmental stresses [25].

Four LEA genes (c32599_g1, c34678_g1, c44015_g1 and c27965_g1) were up-regulated in D_12 data set, and a *LEA* gene (c32475_g1) was down-regulated in D_24 data set. One DEG encoding a seed maturation protein (c32980_g1) which also belongs to LEA family was found to be up-regulated at 12 h. This observation indicated that the *LEA family* gene might be involved in regulating the salinity-resistance in the peanut mutant.

### Identification of Fatty acids biosynthesis and metabolism under salinity stress

Fatty acids are the major components of cell membrane and unsaturated fatty acids play an important role in maintaining the biophysical characteristics of cell membranes. An increased degree of unsaturation increases fluidization of the membrane lipid. Researches have proved that organisms usually maintain the fluidization of membrane by desaturation of the fatty acids to adapt to the environmental changes [26]. In D_6 data set, two DEGs encoding 13-lipoxygenase (c43179_g1and c40135_g1) were down-regulated, while one DEG encoding omega-6 fatty acid desaturase (c35883_g1) and one DEG encoding omega-3 fatty acid desaturase (c36524_g1) were up-regulated. The result showed that the content of unsaturated fatty acids may increase in the salinity-resistant mutant.

These genes which were related to cell wall loosening, LEA, fatty acids biosynthesis and metabolism were also blasted in the database whose access website is (http://www.peanutbase.org), and the related gene and upper promoter sequence had been obtained from *A*. *Duranensis* genome. Related cis-elements in these promoters were analyzed and predicted by Plantcare (http://bioinformatics.psb.ugent.be/webtools/plantcare/html/), and some cis-elements responsive to stress, light and hormone, such as ABRE, ARE, GT1, HSE, MBS, TC-rich repeats and W box were found (S7 File).

In this study, some previously reported stress-related genes have been identified by our DGEs sequencing such as DNA mismatch repair protein MSH6 (c38807_g1), defensin (c1507_g1 and c24576_g1), universal stress protein (c34894_g1), cytochrome P450 (c26974_g1 and c35292_g1), heat shock protein (c33729_g1), low-temperature-induced protein (c42834_g1), metallothionein (c17889_g1), protein phosphatase 2C (c36899_g1), and peroxidase or peroxiredoxin (c13583_g1 and c281633_g1).

Peroxidases are considered bifunctional enzymes that not only oxidize various substrates in the presence of H_2_O_2_, but also generate H_2_O_2_ [27]. Apoplastic peroxidases are known to either restrict or promote cell expansion [28]. The repressed expression of peroxidases by KUA1 (a MYB-like TFs) in leaves promoted cell expansion, which is clearly linked to changed levels of apoplastic H_2_O_2_ [19]. In our study, two DGEs encoding peroxidases were found to be differentially expressed, and one DGE (c13583_g1) was down-regulated and another DGE (c281633_g1) was up-regulated.

These results indicated that the induction or repression of these transcripts in the leaves of peanut under salinity might be necessary for maintaining plant growth under stressful conditions.

Moreover, functional classification showed that 21 unclassified and unknown-function genes out of 166 DEGs, which were responsive to salinity stress, represented the larger set of genes, which may be novel genes involved in salinity stress responses. Among them, the sequences of 12 genes were obtained by blast from *A*. *Duranensis* genome in database http://www.peanutbase.org and these sequence information was listed in S8 File.

## Conclusions

We performed DGE analysis in the leaves of both resistance and susceptibilities genotypes of peanut under the same environmental conditions challenged by salinity stress. The aim of the sequencing was to identify the salinity resistant gene of the HYP-tolerant mutant. The RNA-seq identified candidate TFs and salinity resistance related genes which were involved in change of cell wall structure, accumulation of organic compatible solutes (late embryogenesis abundant protein), ingredient of fatty acid and other known or unknown-function stress related genes.

Collectively, the data indicates that the regulation of salinity stress resistance involves changes in many different aspects which are mentioned above. The information from this study can help us significantly understand the salinity resistant mechanism and provide some important genes resource for peanut breeding.

## Supporting Information

S1 Fig The expression validation of DEGs in leaves of S2 and S4 by real-time PCR using reference genes *Actin*. (TIF)

S2 Fig The expression validation of DEGs in roots of S2 by real-time PCR using reference genes *Actin* and *HDC*. (TIF)

S1 File The summary of DGE library sequencing and analysis. (DOC)

S2 File DEGs between S2 and S4 genotypes at four time points after salinity stress. (XLS)

S3 File Selected DEGs responsive to salinity stress based on comparison of S2 vs. S4. (XLS)

S4 File Result comparison of expression level of 10 DEGs between RNA-seq and real-time PCR. (XLS)

S5 File Difference of relative expression levels of the internal reference genes *Actin*, *TUA5* and *HDC* in leaves and roots after salt stress treatment. (XLS)

S6 File Primers for real-time PCR analysis. (XLS)

S7 File Predictive cis-elements in upper promoter of some genes related to cell wall loosening, LEA, fatty acids biosynthesis and metabolism. (XLSX)

S8 File The basic information of 12 unclassified and unknown-function genes responsive to salinity stress. (DOC)

## Abbreviations

DGE: Digital gene expression
HYP: Hydroxyproline
ABA: Abscisic acid
LEA: Late embryogenesis abundant
HSP: Heat shock protein
TF: Transcription factor
DEG: Differentially expressed gene
XET: Xyloglucan endotransglucosylase/hydrolase
LTP: Lipid transfer protein
SOD: Superoxide dismutase
POD: Peroxidase
MDA: Malondialdehyde
Nr: NCBI non-redundant protein
Nt: NCBI non-redundant nucleotide sequence
SwissProt: The manually annotated and curated protein sequence database
PFAM: Protein family
KOG: EuKaryotic Ortholog Groups
GO: Gene ontology
KEGG: Kyoto Encyclopedia of Genes and Genomes
