# Supplementary figures and images for: The Salinity Responsive Mechanism of a Hydroxyproline-Tolerant Mutant of Peanut Based on Digital Gene Expression Profiling Analysis
Source: PLoS One. 2016 Sep 23;11(9):e0162556. doi: 10.1371/journal.pone.0162556 (PMC5035014; doi:10.1371/journal.pone.0162556)

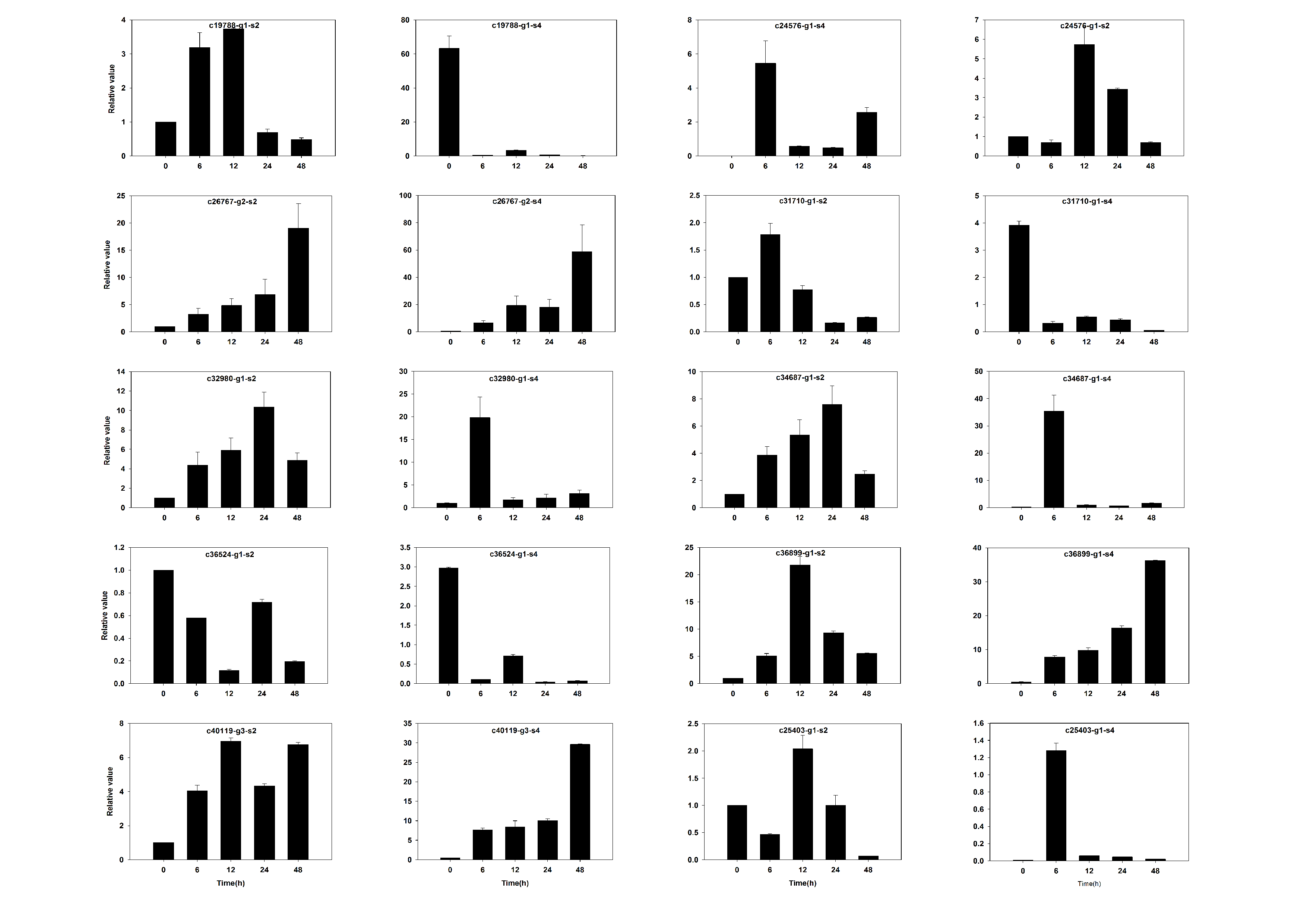

Supplement: S1 Fig — (TIF) [file pone.0162556.s001.tif]

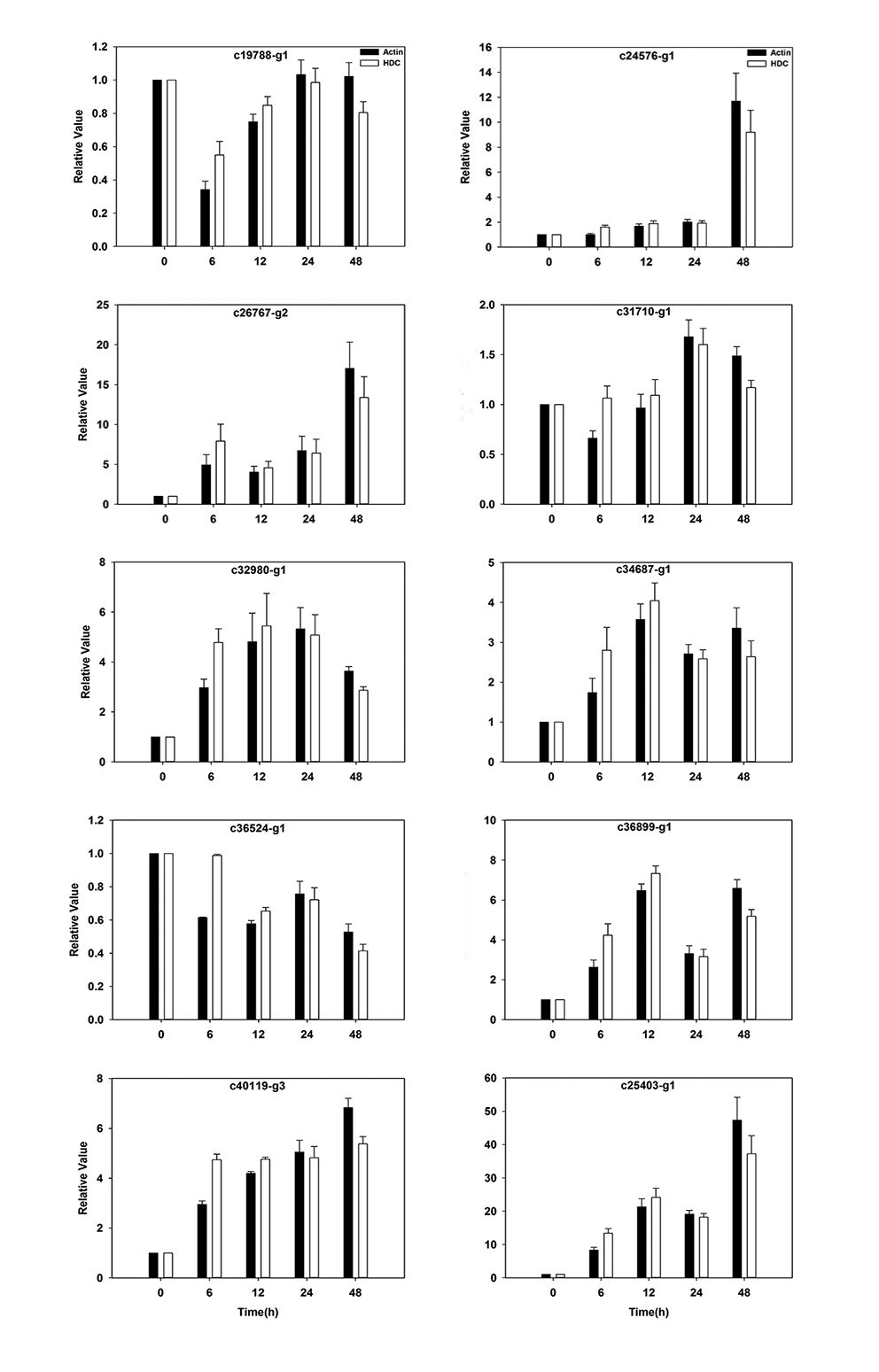

Supplement: S2 Fig — (TIF) [file pone.0162556.s002.tif]
