# Supplementary material for: The Salinity Responsive Mechanism of a Hydroxyproline-Tolerant Mutant of Peanut Based on Digital Gene Expression Profiling Analysis
Source: PLoS One. 2016 Sep 23;11(9):e0162556. doi: 10.1371/journal.pone.0162556 (PMC5035014; doi:10.1371/journal.pone.0162556)
Supplement: S1 File — (DOC) [file pone.0162556.s003.doc]

| Samples | Raw Reads | Clean Reads | Clean Bases | Total mapped Reads | Total mapped reads/Clean reads (%) | Q20 (%) | Q30 (%) |
| --- | --- | --- | --- | --- | --- | --- | --- |
| S2_0_1 | 13130522 | 13064537 | 0.65G | 11867259 | 90.84 | 98.72 | 95.59 |
| S2_0_2 | 12372949 | 12154348 | 0.61G | 11041136 | 90.84 | 98.71 | 95.61 |
| S4_0_1 | 15056177 | 14924989 | 0.75G | 13455083 | 90.15 | 97.96 | 93.60 |
| S4_0_2 | 13676909 | 13456449 | 0.67G | 12092554 | 89.86 | 97.87 | 93.34 |
| S2_6_1 | 11925146 | 11657894 | 0.58G | 10527096 | 90.30 | 98.74 | 95.66 |
| S2_6_2 | 11882160 | 11597012 | 0.58G | 10471859 | 90.30 | 98.74 | 95.63 |
| S4_6_1 | 15578928 | 15301563 | 0.77G | 13828909 | 90.38 | 97.91 | 93.49 |
| S4_6_2 | 13000125 | 12817785 | 0.64G | 11545930 | 90.08 | 97.94 | 93.56 |
| S2_12_1 | 10918744 | 10619349 | 0.53G | 9561483 | 90.04 | 98.63 | 95.43 |
| S2_12_2 | 11607913 | 11468361 | 0.57G | 10302785 | 89.84 | 98.58 | 95.18 |
| S4_12_1 | 12592347 | 12349251 | 0.62G | 11128758 | 90.12 | 97.85 | 93.34 |
| S4_12_2 | 13979107 | 13865925 | 0.69G | 12463092 | 89.88 | 97.90 | 93.47 |
| S2_24_1 | 11925684 | 11767778 | 0.59G | 10635781 | 90.38 | 98.71 | 95.60 |
| S2_24_2 | 11946263 | 11467867 | 0.57G | 10309771 | 89.90 | 98.64 | 95.47 |
| S4_24_1 | 11344406 | 11225546 | 0.56G | 10082621 | 89.82 | 97.85 | 93.36 |
| S4_24_2 | 10553695 | 10404324 | 0.52G | 9347520 | 89.84 | 97.86 | 93.35 |
| S2_48_1 | 10818494 | 10622732 | 0.53G | 9546329 | 89.87 | 98.65 | 95.46 |
| S2_48_2 | 10548973 | 10407653 | 0.52G | 9233893 | 88.72 | 98.51 | 95.16 |
| S4_48_1 | 13152121 | 13070881 | 0.65G | 11791177 | 90.21 | 97.91 | 93.47 |
| S4_48_2 | 12323387 | 12153629 | 0.61G | 10926693 | 89.90 | 97.85 | 93.34 |
