# Supplementary material for: The Salinity Responsive Mechanism of a Hydroxyproline-Tolerant Mutant of Peanut Based on Digital Gene Expression Profiling Analysis
Source: PLoS One. 2016 Sep 23;11(9):e0162556. doi: 10.1371/journal.pone.0162556 (PMC5035014; doi:10.1371/journal.pone.0162556)
Supplement: S8 File — (DOC) [file pone.0162556.s010.doc]

| gene_id | Chromosome  localization | Subcellular  localization | Protein  size (aa) | Molecular  Weigh (kDa) | Isoelectric  point | Number of exons |
| --- | --- | --- | --- | --- | --- | --- |
| c550_g1 | 1 | nucleus | 367 | 40.43 | 8.24 | 3 |
| c3279_g1 | 6 | mitochondrion | 523 | **59.01** | 7.19 | 9 |
| c12384_g1 | 6 | cytoplasm | 177 | 18.08 | 8.19 | 2 |
| c25653_g1 | 5 | nucleus | 55 | 6.40 | 9.75 | 2 |
| c26926_g1 | 10 | endoplasmic reticulun | 363 | 39.87 | 9.31 | 7 |
| c29473_g1 | 8 | extracellular | 179 | 20.48 | 10.08 | 2 |
| c33038_g1 | 7 | endoplasmic reticulun | 114 | 10.92 | 5.70 | 4 |
| c35384_g1 | 4 | cytoplasm | 179 | 19.36 | 6.78 | 2 |
| c36641_g2 | 3 | mitochondrion | 411 | 47.32 | 9.27 | 10 |
| c53991_g1 | 3 | chloroplast | 399 | 43.10 | 9.23 | 5 |
| c60993_g1 | 6 | cell wall | 487 | 53.88 | 5.47 | 9 |
| c61312_g1 | 5 | mitochondrion | 167 | 17.82 | 5.42 | 4 |
